# A Dual-Channel Enhanced Mamba Model for Fault Detection in Grid-Connected Photovoltaic Systems

**DOI:** 10.3390/s26123764

**Published:** 2026-06-12

**Authors:** Yu Zhu, Qiang Yang

**Affiliations:** College of Electrical Engineering, Zhejiang University, Hangzhou 310027, China; zhuyu_ee2022@zju.edu.cn

**Keywords:** photovoltaic systems, time-series data, fault detection

## Abstract

Accurate fault detection is essential for the safe and reliable operation of grid-connected photovoltaic (PV) systems under complex and dynamically varying conditions. However, existing data-driven approaches are often hindered by the scarcity of labeled fault data and by their limited ability to model complex multivariate temporal dependencies. To address these challenges, this paper first develops a realistic simulation of a grid-connected PV system to generate a large volume of labeled multivariate time-series fault data spanning diverse fault scenarios under varying operating conditions. The simulated data augment the limited real-world measurements, improving fault coverage and model generalization. On this basis, a dual-channel enhanced Mamba model is proposed for PV fault detection. The model decouples temporal modeling and variable-wise modeling into two dedicated channels, enabling complementary extraction of global temporal dependencies and intra-variable dynamics. Extensive experiments show that the proposed approach consistently outperforms several mainstream time-series classification methods in accuracy, precision, recall, and F1-score, demonstrating that it provides an effective and scalable solution for data-driven fault detection in grid-connected PV systems.

## 1. Introduction

Photovoltaic (PV) systems have been widely adopted as a clean and renewable energy source, contributing to the global transition towards low-carbon power generation [1,2]. However, practical PV plants consist of numerous interacting components and control loops, making them vulnerable to anomalies and faults that can reduce energy yield and pose operational and safety risks [3]. These faults arise on both the DC and the AC side of the system, and their consequences differ markedly. On the DC side, faults such as string open-circuit and short-circuit conditions, partial shading, and current-sensor errors directly reduce the power output of the affected strings and distort the array’s operating point; severe DC-side faults, in particular short-circuit and ground faults, generate sustained high currents and may trigger arc formation, posing a significant fire risk [4]. On the AC side, inverter faults (e.g., failure of the IGBT switches) and grid anomalies such as voltage sags and frequency deviations degrade the quality of the injected power and, in severe cases, threaten the stability of the point of common coupling and the connected grid [5]. Moreover, faults frequently occur in combination, for example, partial shading together with an open-circuit fault, or a grid anomaly concurrent with an inverter fault, producing compound signatures that are even harder to identify. The strong coupling between DC- and AC-side measurements, the multivariate and time-varying nature of the signals, and the presence of such compound faults make accurate and timely fault identification in PV systems particularly challenging. Therefore, reliable real-time fault and anomaly detection is critical to maintaining the efficiency, availability, and safe operation of PV plants.

The detection of faults and anomalies in PV systems presents several significant challenges due to the complex and dynamic nature of these systems. One major challenge is the variation in operating conditions, such as fluctuating irradiance and temperature, which cause shifts in system performance over time. These environmental factors lead to changes in the electrical characteristics of the system, making it difficult to distinguish between normal operational variations and actual faults [6]. Additionally, the fast and transient nature of certain faults, such as short circuits or grid disturbances, results in weak or brief abnormal signals that are challenging to detect. The impact of control strategies, such as Maximum Power Point Tracking (MPPT), further complicates the detection process. While MPPT optimizes power output, it can mask fault symptoms by continuously adjusting the operating points, especially when faults occur during MPPT operation [7]. Moreover, real-world measurement data often contains noise and uncertainty, including sensor inaccuracies, communication delays, and synchronization issues, which can degrade the performance of fault detection algorithms. Finally, the lack of labeled fault data in many PV plants limits the effectiveness of machine learning models, necessitating the use of unsupervised or semi-supervised learning techniques, which adds another layer of complexity [6,8]. These challenges underscore the need for adaptive, robust fault detection systems that can effectively handle the variability, complexity, and uncertainties inherent in PV system operation.

Fault detection in photovoltaic (PV) systems has been extensively studied. The methods range from conventional model-based techniques to more recent data-driven approaches, with a particular focus on leveraging machine learning and deep learning [9,10]. Model-based approaches are grounded in the mathematical modeling of PV systems and focus on comparing the actual performance with predicted outputs to detect anomalies. The authors in [11] proposed a fault-locating strategy for large-scale PV arrays that uses voltage sensor deployment and mathematical modeling to identify faulty module blocks. These methods are based on the physical behavior of PV systems but can be limited by the complexity of modeling real-world conditions. The work in [12] proposed a fault detection and location algorithm for photovoltaic systems that uses voltage characteristics, based on Kirchhoff’s Voltage Law, to detect line-to-ground and line-to-line faults, even under varying fault impedance and partial shading conditions. The work in [13] proposed a model-based fault detection method for PV arrays that uses differential voltage measurements between adjacent string modules and optimized sensor placement to detect and localize faults, providing a cost-effective and reliable solution even in systems with blocking diodes and varying fault conditions.

Data-driven approaches, particularly machine learning models, have gained popularity due to their ability to handle high-dimensional, time-series data and detect complex fault patterns without requiring explicit system models. Comprehensive evaluations of machine learning and ensemble-based fault detection methods have demonstrated their effectiveness across a wide range of PV fault scenarios [14]. The work in [15] employed deep recurrent neural networks (RNNs) for fault diagnosis in PV systems, focusing on uncertainty and real-time detection in uncertain environments. Similarly, statistical data-driven methods have also been explored. The work in [16] developed a data-driven anomaly detection method using Kernel Density Estimation (KDE) to model the output characteristics of PV modules, enabling the identification of abnormal modules based on their probability distribution features. While related statistical monitoring approaches have demonstrated robustness under measurement noise and operating uncertainties [17]. The authors in [18] presented a method for fault detection and diagnosis in photovoltaic systems, using Autoencoder Neural Networks (AEs) for unsupervised feature extraction and Deep Neural Networks (DNNs) for fault classification, improving detection accuracy even with noisy data. These methods, relying on historical data to identify deviations from normal behavior, are often more adaptable than conventional model-based methods but still require high-quality data, which may not always be available.

The development of fault-detection methods has progressed through several stages, each motivated by the limitations of the previous one: from model-based approaches that depend on accurate physical models, to classical machine-learning methods relying on hand-crafted features, and further to deep-learning methods that extract features automatically [19,20]. A persistent challenge across these stages is the scarcity of labeled fault data, which has motivated several recent strategies. The work in [21] proposed a self-supervised time–frequency dual-domain prediction (TFDDP) framework that learns fault representations from large amounts of unlabeled bearing data, while the authors in [22] developed a digital twin-assisted graph contrastive domain-adaptation (GCDA) network, in which a physics-based dynamic model generates simulated data whose distribution is explicitly aligned with measured signals. These works show that combining physics-based simulation or self-supervised learning with deep models is an effective route to alleviating label scarcity, a principle that also underlies the present work. However, these methods mainly target bearing or rotating-machinery diagnosis, whereas the present work addresses multivariate fault classification in grid-connected PV systems, where the key difficulty lies in jointly modeling global temporal dependencies and inter-variable correlations among heterogeneous DC- and AC-side measurements. To this end, a high-fidelity PV simulation is used to augment limited real measurements, and a Mamba-based dual-channel architecture is adopted to capture both dependency types at near-linear cost, avoiding the O(L^2^) complexity of Transformer-based encoders.

Challenges persist in ensuring real-time fault detection, maintaining high accuracy under changing environmental conditions, and ensuring cost-effectiveness. Recent studies have explored edge-based fault detection frameworks to reduce latency and improve real-time responsiveness, highlighting the importance of system-level design in practical PV fault diagnosis [23]. Many existing solutions rely on high-frequency sensors, which can be expensive and difficult to deploy at scale. To alleviate deployment and monitoring constraints, machine-learning-based fault diagnosis has been integrated with IoT-enabled remote monitoring architectures, enabling online fault detection with reduced sensing and communication overhead [24]. Additionally, fault detection in PV systems under MPPT (Maximum Power Point Tracking) conditions remains challenging, as MPPT can mask fault symptoms by continuously adjusting the system’s operating point. To address these challenges, the authors in [25] developed a real-time fault detection system that combines multi-sensor data with Principal Component Analysis (PCA) and Kernel Density Estimation (KDE), enabling accurate fault detection in PV systems under MPPT conditions while reducing computational complexity. In a similar context, the work in [26] applied Deep Recurrent Neural Networks (DRNNs) to provide real-time fault diagnosis in uncertain PV systems, showing strong adaptability to environmental variations. Additionally, the study in [27] proposed a method using Variational Autoencoders (VAE) for automatic anomaly detection in grid-connected PV systems, highlighting its effectiveness even with limited labeled data.

While significant advancements have been made in fault detection for photovoltaic (PV) systems, several critical challenges remain that limit the effectiveness of existing methods. One major challenge is the high-dimensional, correlated data collected from multiple sensors, which can significantly complicate the decision-making process. The high correlation between variables from the DC and AC sides of PV systems increases the complexity of feature extraction, making it harder to identify meaningful patterns for fault detection. Furthermore, sensor data often suffers from issues such as missing observations due to varying sampling rates or random data loss, especially at specific time points, which creates gaps in the dataset and reduces the overall reliability of the fault detection system [26]. This is particularly problematic in large-scale PV systems where the continuous and real-time monitoring of all components is essential for maintaining performance and safety. Another challenge is the imbalanced nature of fault data, with certain faults occurring rarely, particularly under extreme environmental conditions [28]. This imbalance leads to insufficient fault data for training deep learning models, limiting the generalizability of fault detection systems to rare fault scenarios. Moreover, non-uniform data distribution further complicates the training process by introducing inconsistencies across data sources. These issues are compounded by the inherent uncertainty in PV systems, where external factors such as temperature and irradiance fluctuations affect the system’s behavior, making it difficult to model and predict faults under varying conditions.

In summary, the existing solutions either highly rely on idealized models or require large and labeled datasets to perform effectively. Furthermore, many solutions are considered computationally expensive and struggle to meet the real-time demands of PV system monitoring. As a result, there is a pressing need for adaptive and efficient algorithms that can handle high-dimensional, noisy, and imbalanced data, while providing reliable and real-time fault detection in PV systems.

This work aims to fill these gaps by proposing an improved time-series deep learning method that integrates both DC and AC-side measurements to detect faults in real time, even under uncertain and dynamically changing conditions. By leveraging simulation data across various fault scenarios and addressing the challenges of data imbalance and uncertainty, this study aims to develop a more robust, scalable, and efficient fault detection system for PV systems. The main technical contributions of this work can be summarized as follows:

1. This work constructs a hybrid multivariate time-series dataset by augmenting real-world measurements with simulated fault scenarios generated from a comprehensive PV system model. The resulting dataset is made publicly available to support research on real-time PV fault diagnosis and serve as a standardized benchmark for data-driven monitoring.
2. To improve the fault detection performance in grid-connected PV systems, a Dual-Channel Enhanced Mamba Model is proposed that simultaneously captures both local dependencies within variables and global frequency-domain dependencies. This model is specifically designed to handle the multivariate dependency scenarios typical of grid-connected PV systems, enabling more accurate fault classification and better performance in detecting faults across diverse operating conditions.

The structure of the paper is as follows: Section 2 presents the proposed method, Section 3 discusses the experiments and results, Section 4 analyzes the ablation experiments, and Section 5 concludes with a discussion of future work.

## 2. Materials and Methods

### 2.1. Preliminaries: State-Space Models and Mamba

(1) State-Space Models: A continuous-time SSM [29] describes the evolution of a system through a latent state, mapping an input signal x(t)∈ℝ to an output y(t)∈ℝ via an N-dimensional hidden state $h(t)\in {\mathbb{R}}^{N}$:

(1) $$h^{\prime }(t)=Ah(t)+Bx(t),y(t)=Ch(t)$$

where $A\in {\mathbb{R}}^{N\times N}$ is the state transition matrix, and $B\in {\mathbb{R}}^{N\times 1}$ and $C\in {\mathbb{R}}^{1\times N}$ are the input and output projection matrices, respectively. Since measurement sequences in PV systems are sampled at discrete intervals, the continuous system in Equation (1) is discretized with a timescale parameter Δ using the zero-order hold (ZOH) rule:

(2) $$\bar{A}=\exp(\Delta A),\bar{B}={\left(\Delta A\right)}^{-1}(\exp(\Delta A)-I)\Delta B$$

which yields the discrete-time recurrence

(3) $$h_{k}=\bar{A}h_{k-1}+\bar{B}x_{k},y_{k}=Ch_{k}$$

Equation (3) can be computed recurrently with O(L) complexity for a sequence of length *L*. Because the recurrence is linear, it can also be unrolled into an equivalent global convolution, which permits parallel training. The structured SSM (S4) further imposes a HiPPO-based structure on *A* to strengthen long-range dependency modeling. However, in S4 the parameters ($\bar{A}$, $\bar{B}$, C, Δ) are fixed and shared across all time steps, making the model time-invariant and content-agnostic; it cannot selectively emphasize or suppress information according to the input, which is a limitation when fault signatures are transient and weak [30].

(2) The Mamba Architecture: Mamba overcomes this limitation by introducing a selective mechanism [31], in which the input and output projections B, C, and the timescale Δ are made functions of the current input:

(4) $$B_{k}=Linear_{B}(x_{k}),C_{k}=Linear_{C}(x_{k}),\Delta_{k}=softplus(Linear_{\Delta }(x_{k}))$$

Through this input-dependent parameterization, Mamba dynamically modulates how each time step interacts with the latent state, allowing it to retain salient fault-related transients while filtering out routine operational fluctuations. A hardware-aware parallel scan algorithm preserves training efficiency, so that Mamba maintains near-linear O(L) complexity in the sequence length.

PV monitoring produces long multivariate sequences with high sampling rates. Transformer-based encoders model such dependencies via self-attention at O(L^2^) complexity, which grows prohibitively with sequence length and variable count. Mamba achieves comparable representational capacity at near-linear cost, making it well-suited for real-time PV fault detection.

### 2.2. Grid-Connected PV System

**Figure 1:**
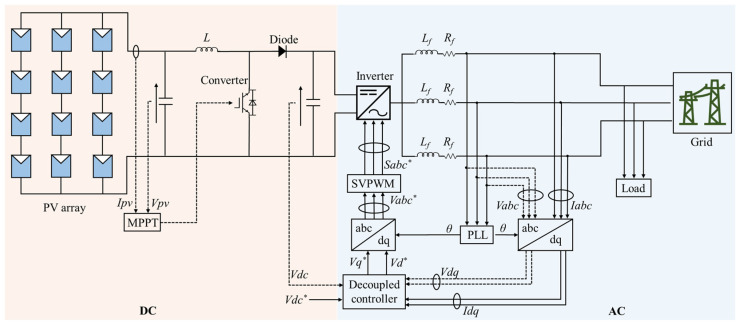
Schematic overview of the simulated grid-connected PV system. Solid lines represent electrical connections, while dashed lines represent control and measurement signal paths. Variables with the superscript “*” denote their reference values.

This section focuses on the development of a simulation model for the grid-connected PV system, designed to generate a large amount of fault time-series data for use in the fault detection model presented in the next section. The typical schematic diagram of a grid-connected photovoltaic system, equipped with conventional protection devices and the proposed sensor layout, is shown in Figure 1.

The system comprises multiple interconnected components, including the PV array, Maximum Power Point Tracking (MPPT) controller, inverter, and grid interface. The PV array is configured with three parallel strings, with each string comprising seventeen PV modules connected in series. The configuration parameters of each PV module are listed in Table 1. The control structure employs Voltage-Oriented Control (VOC) combined with Space Vector Pulse Width Modulation (SVPWM) to manage active and reactive power, ensuring synchronization with the grid through a Phase-Locked Loop (PLL). The inverter’s output is connected to the grid, allowing for power transfer between the PV system and the grid. A total of nine variables are measured in the grid-connected PV system, including the DC-link voltage $V_{dc}$, the PV-side voltage $V_{pv}$ and current $I_{pv}$, and the three-phase voltages $V_{abc}$ and three-phase currents $I_{abc}$ at the AC side of the inverter.

The nonlinear and time-varying characteristics of PV systems can be theoretically described using the ideal one-diode model [32], which establishes a relationship between the output voltage $V_{pv}$ and the output current $I_{pv}$:

(5) $$I_{pv}=I_{ir}-I_{0}\left[\exp\left(\frac{V_{pv}+R_{s}I_{pv}}{\alpha V_{th}}\right)-1\right]-\frac{V_{pv}+R_{s}I_{pv}}{R_{sh}}$$

where $V_{pv}$ and $I_{pv}$ are the output voltage and current of the module, respectively; $I_{ir}$ is the photocurrent of the module; $I_{0}$ is the reverse saturation current of the diodes of PV cells constituting the module; α is the diodes’ ideality factor; $V_{th}$ is the thermal voltage, $R_{s}$ and $R_{sh}$ are the series and parallel equivalent resistance of the module, respectively. The system is nonlinear and time-varying because the diode saturation current $I_{0}$ is a function of the cell temperature T, while the photocurrent $I_{ir}$ depends on the irradiance level G and also varies with T.

In the simulated grid-connected PV system, a total of nine electrical quantities are measured for fault diagnosis. Specifically, the measured signals include the PV-side voltage $V_{pv}$ and current $I_{pv}$, the DC-link voltage $V_{dc}$, the three-phase grid voltages $V_{abc}$, and the three-phase grid currents $I_{abc}$. The sampling time for each data point is set at 100 μs. These measurements are organized into a data metric as follows.

(6) $$X=\left[V_{pv},I_{pv},V_{dc},V_{a},V_{b},V_{c},I_{a},I_{b},I_{c}\right]$$

Inspired by the fault classification in [19], six distinct fault scenarios are investigated, as summarized in Table 2. Each fault type is varied in terms of location, severity, and system topology to ensure a comprehensive analysis. Each fault is manually imposed in an independent simulation run lasting 5–10 s, with the fault initiated around the 2nd–4th s. From the continuous signal of each run, a sliding window of 5000 consecutive sampling points, corresponding to 0.5 s at the 100 μs sampling interval, is extracted to form one sample, so that the system performs one fault diagnosis every 0.5 s. Each window is positioned to cover the transition from normal to faulty operation, so that the fault onset appears at an intermediate position within the 5000-point sample. The dataset consists of one normal class and eight fault classes, totaling nine categories. Each category contains 500 samples, resulting in a total of 4500 samples.

The open-circuit fault refers to a break in the electrical connections of the PV system, which can occur due to detached cables or melting caused by a short circuit. In this study, two fault configurations are considered: the disconnection of one or two substrings. A short-circuit fault in the PV system can affect individual PV cells, bypass diodes, or entire modules. Short circuits can result in energy losses, fire hazards, and electric shock risks [10]. For the simulation, different levels of short-circuit severity are considered, including short circuits in individual modules, combinations of modules, and inter-string short circuits. Considering the randomness of fault occurrences under actual operating conditions, various fault resistances and capacitances are configured in the simulation.

Sensor faults typically occur due to issues such as poor connections, signal distortion, or hardware malfunctions, which result in inaccuracies in the measurement data. These faults often lead to the misinterpretation of critical system parameters, such as voltage and current, thereby affecting the accuracy of system control and monitoring [33]. During the simulation, sensor faults are simulated by introducing predefined errors into the sensor outputs to represent hardware malfunctions. These errors are randomly configured within a range of 5% to 30% of the original readings.

Partial shading occurs when parts of the PV modules receive less irradiance than others. This can be caused by passing clouds, tall buildings, trees, chimneys, or other obstacles. Extended partial shading may lead to hot spots, which are localized power dissipation areas caused by reverse bias across shaded PV cells [34,35]. Four severity levels of partial shading are simulated by reducing irradiance on the following number of modules: a 20–30% reduction on one module, and a 10–20% reduction on four modules.

However, such faults typically have a mild impact, resulting in only minor power losses without significantly affecting system performance. In contrast, inverter faults and grid anomalies occurring on the AC side are more easily detectable, as the data from the AC side exhibits minimal variability, as shown in the simulation results. Despite being easier to detect, these faults are more severe due to their direct impact on the system’s power quality and overall stability [36]. The inverter faults are set to trigger at different severities, such as the complete failure of one to three IGBTs. Additionally, grid anomalies are simulated by introducing disturbances such as voltage sag, frequency deviations, or phase imbalances, which are randomly applied at various time intervals. In practical PV systems, multiple faults may occur simultaneously, forming concurrent fault conditions that significantly increase the complexity of fault diagnosis [37].

### 2.3. Dual-Channel Enhanced Mamba Model (DCEM)

Grid-connected photovoltaic (PV) systems are inherently dynamic and subject to a wide range of operational uncertainties due to fluctuating environmental conditions, intermittent generation, and the occurrence of various electrical faults. In practical operation, system measurements such as DC-side voltages and currents, AC-side waveforms, and converter signals exhibit complex temporal patterns that reflect both normal behavior and multiple classes of fault conditions [38]. Accurate fault diagnosis in such systems, therefore, requires not only sensitivity to subtle changes in these signals, but also the ability to capture long-term temporal dependencies embedded in the measurement sequences. Conventional pattern recognition and shallow learning methods often struggle to generalize across diverse fault types and varying operating conditions because they fail to exploit the temporal context within long measurement histories fully [39]. Against this backdrop, a modeling approach that effectively accommodates the long-range dependencies and rich temporal structure inherent in PV system signals is essential for enhancing the reliability and accuracy of automated fault diagnosis.

**Figure 2:**
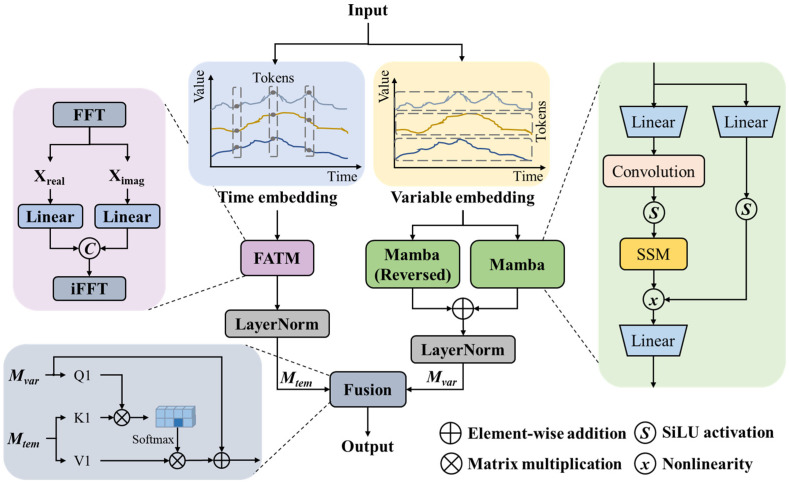
Structural diagram of the proposed dual-channel enhanced mamba model. Solid black lines indicate the main data/module connections, dashed black lines indicate enlarged views or module correspondence, and arrows indicate the data-flow direction. The colored curves are used to distinguish different variables.

The method presented in this paper employs a dual-channel architecture, designed to simultaneously capture and model both temporal and spatial dependencies within multivariate time-series data from grid-connected PV systems. As illustrated in Figure 2, the framework comprises distinct channels that process data from two complementary perspectives: time embedding and variable embedding.

(1) Time Embedding Channel: The raw input data $X\in R^{B\times L\times V}$, where *B* is the batch size, *L* is the length of the time series, and *V* is the number of variables, is first mapped into a D-dimensional feature space by a 1-D convolutional token embedding, producing $X_{tem}\in R^{B\times L\times D}$. To preserve the order of time steps, a sinusoidal positional encoding $PE\in R^{L\times D}$ is then added to X*_tem_* defined element-wise as

(7) $$PE_{\left(l,2i\right)}=\sin(l/10000^{2i/D}),PE_{\left(l,2i+1\right)}=\cos(l/10000^{2i/D})$$

where $l\in \left\{0,\ldots ,L-1\right\}$ is the time-step index and $i\in \left\{0,\ldots ,\frac{D}{2}-1\right\}$ is the feature-dimension index. The temporal embedding token X*’_tem_* is obtained by the tensor addition

(8) $${X^{\prime }}_{tem}=X_{tem}+PE$$

The positional encoding ensures that the temporal relationships between time steps are preserved. This temporal embedding captures the instantaneous correlations among the variables at each time step, which is essential for the encoder to extract global temporal dependencies. The frequency-domain transformation is applied to the input data to extract periodic features and better capture temporal dependencies. The embedded time-series data is then transformed into the frequency domain using Fast Fourier Transform (FFT) along the temporal dimension. The real and imaginary parts of the FFT result are extracted separately and concatenated. The embedding data undergoes a Frequency-Aware Temporal Module (FATM) process to extract global temporal dependencies. The output features are processed through a linear transformation to ensure stable learning and efficient feature integration. Next, the real and imaginary parts of the frequency-domain data are split, then combined into a complex tensor. Finally, an Inverse FFT (iFFT) is applied to the complex tensor to transform the data back into the time domain, yielding the final output. The FFT is adopted here for its efficiency and exact invertibility, which allows the spectrum to be processed and then reconstructed back to the time domain. Both the real and imaginary parts are retained so that not only the magnitude but also the phase relationships among frequency components, which are altered by faults such as inverter faults and grid anomalies, are preserved for discrimination.

(2) Variable Embedding Channel: This step in the embedding process involves transposing the raw input data $X\in R^{B\times L\times V}$ to $X^{T}\in R^{B\times V\times L}$. Subsequently, a linear layer is employed to map the temporal dimension *L* to a high-dimensional feature space *D*, thereby generating the variable embedding token $X_{\mathrm{var}}\in R^{B\times V\times D}$. This mapping allows the model to capture the temporal dependencies for each variable, with each variable’s individual features embedded in a shared D-dimensional space before further processing for inter-variable interactions. No positional encoding is applied in this channel, since the measured variables have no inherent ordering. After the variable embeddings are created, the data is passed through the Encoder, where bidirectional Mamba [31] is employed to model the local temporal dependencies within each variable. This allows the model to learn the dependencies between time steps in both causal (forward) and anti-causal (reverse) directions, ensuring a comprehensive understanding of the temporal dynamics for each variable [40]. Given the input sequence *X*, the forward Mamba processes the sequence in the natural order, updating the state stf using the state-space model as:

(9) $$s_{t}^{f}=A^{f}s_{t-1}^{f}+B^{f}X_{t}$$

where *A^f^* and *B^f^* are the state transition matrices for the forward pass. Similarly, the reverse Mamba processes the sequence in reverse order, updating the state str as:

(10) $$s_{t}^{r}=A^{r}s_{t+1}^{r}+B^{r}X_{t}$$

where *A^r^* and *B^r^* are the state transition matrices for the reverse pass. The outputs from both the forward and reverse Mamba blocks are then combined to produce the final bidirectional output at each time step:

(11) $$Y_{t}=s_{t}^{f}+s_{t}^{r}$$

This bidirectional processing enhances the model’s ability to learn from both past and future time steps, improving its understanding of the temporal dynamics of each variable and leading to better feature extraction for tasks such as forecasting and fault diagnosis.

(3) Fusion Strategy: The fusion module aligns temporal and variable features by first projecting the temporal tokens to match the variable dimension using a cross-attention mechanism. Variable tokens *M*_var_ act as queries, while temporal tokens *M_tem_* serve as keys and values. This design allows each variable token to selectively attend to relevant temporal patterns, rather than relying on a fixed temporal-to-variable projection. Before attention computation, layer normalization is applied to both inputs to enhance training stability. The cross-attention output captures variable-conditioned temporal context and is further processed by a lightweight feed-forward network to refine the auxiliary features. To prevent the auxiliary temporal information from overwhelming the primary variable representation, a variable-dependent gating mechanism is introduced. The gate values are computed from the original variable representation and passed through a sigmoid function, yielding a modulation factor for each variable token. Finally, the fused representation is obtained via a gated residual injection, formulated as

(12) $$M_{out}=M_{\mathrm{var}}+\alpha \cdot attn(M_{\mathrm{var}},M_{tem})$$

where *M*_var_ denotes the variable-wise representation, *M_tem_* denotes the temporal-wise representation, *α* is a learnable scaling parameter, *attn* denotes the cross-attention mechanism. *M*_var_ forms the backbone of the fused representation, while M_temporal is injected as a variable-conditioned, gated complementary term whose strength is adaptively controlled by the learnable scalar *α*.

By explicitly modeling variable-guided temporal attention and conservative residual injection, the proposed fusion module effectively captures both intra-variable dependencies and cross-temporal correlations, making it suitable for multivariate time-series analysis in grid-connected photovoltaic systems.

## 3. Experiment and Analysis

### 3.1. Experimental Setup

(1) Dataset

**Figure 3:**
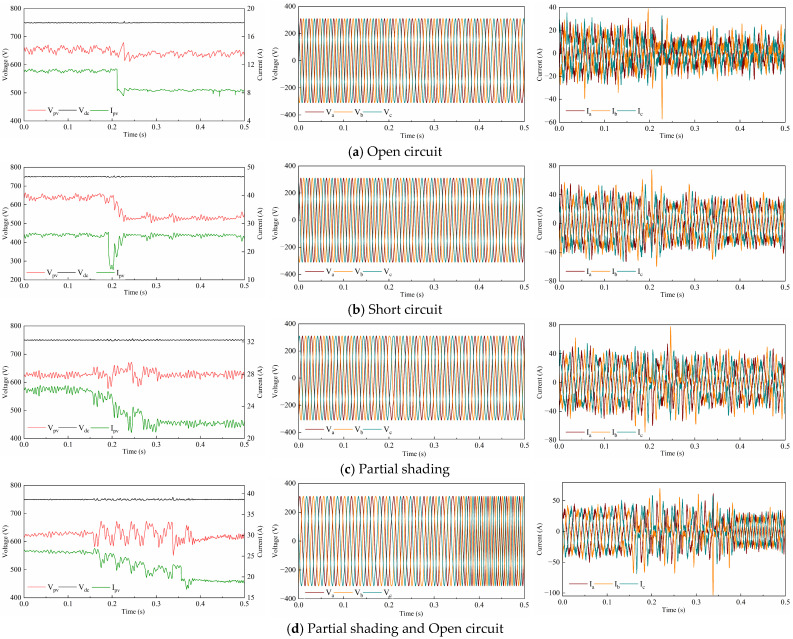

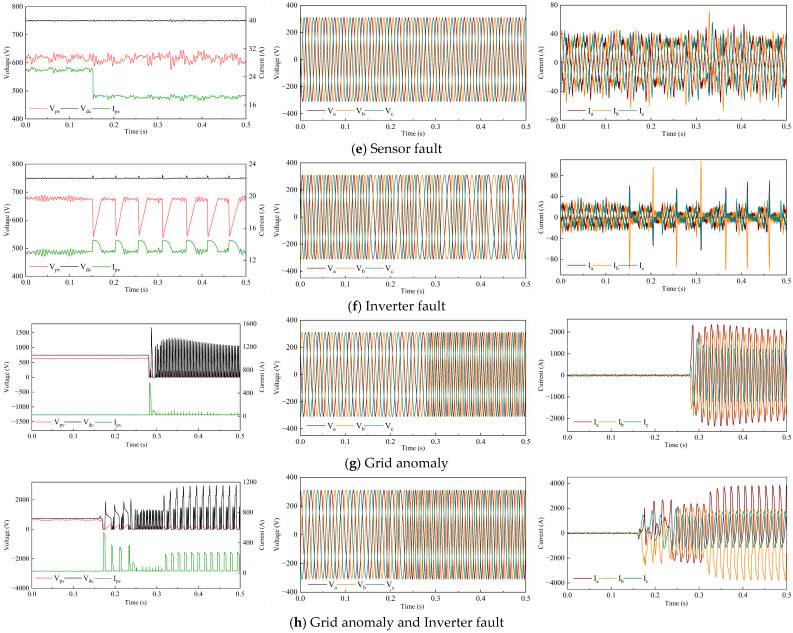
Representative time-series signals of different fault categories in the dataset. The horizontal axis denotes the 5000 sampling points (0.5 s) of one extracted sample window.

To improve the generalizability and practical relevance of the proposed fault diagnosis method, this study constructs a hybrid dataset composed of both simulated and real-world measurements. The simulated portion, accounting for 80% of the data, is generated based on a high-fidelity Simulink model of a grid-connected PV system, with fault scenarios and measurement variables detailed in Section 2.1. Each configuration is based on two years of publicly available meteorological data, which is used to determine the irradiance and temperature of the photovoltaic (PV) modules. The real-world data, constituting the remaining 20%, are collected from 36 operational grid-connected PV power plants over a continuous period of one month. These plants are equipped with standard monitoring systems that record PV-side voltage and current, DC-link voltage, and three-phase AC-side signals at regular sampling intervals. A total of 900 real data samples were extracted and manually verified, covering all eight fault categories described in this paper. All real-world signals were collected at a fixed sampling rate and then aligned with simulated data for unified preprocessing. The overall dataset includes eight fault types, as detailed in Table 1, along with a normal category, resulting in a total of nine categories. The dataset comprises nine categories, one normal class, and eight fault classes, with 500 samples per category, resulting in 4500 samples in total. Each sample is a 5000 × 9 matrix, where 5000 denotes the number of time steps in one sample (a 0.5 s window sampled at 100 μs, see Section 2.1) and 9 denotes the number of monitored variables. The complete dataset, therefore, has the shape 4500 × 5000 × 9 (samples × time steps × variables). As shown in Figure 3, the time-series signals corresponding to different fault categories exhibit diverse and overlapping temporal patterns, which makes accurate fault diagnosis particularly challenging. The dataset is partitioned such that the model is trained on the simulated fault data and evaluated on a test set composed entirely of real-world measurements. The dataset produced in this work is available at https://github.com/ZzzYyy0614/PVsystems_datasets (accessed on 29 March 2026) to facilitate reproducibility and further research.

(2) Evaluation metrics

To thoroughly evaluate the model’s performance, we utilized classification accuracy, precision, recall, and *F*1 score as evaluation metrics, which are defined in Equation (13) to Equation (16).

(13) $$Precision_{macro}=\frac{1}{C}\sum_{i=1}^{C}\frac{TP_{i}}{TP_{i}+FP_{i}}$$

(14) $$Recall_{macro}=\frac{1}{C}\sum_{i=1}^{C}\frac{TP_{i}}{TP_{i}+FN_{i}}$$

(15) $$F1_{macro}=\frac{1}{C}\sum_{i=1}^{C}\frac{2TP_{i}}{2TP_{i}+FP_{i}+FN_{i}}$$

(16) $$Accuracy_{macro}=\frac{\sum_{i=1}^{C}TP_{i}+TN_{i}}{N}$$

where *TP* (true positives) presents the number of samples that are correctly classified as positive, *TN* (true negatives) presents the number of samples that are correctly classified as negative, *FP* (false positives) presents the number of samples that are incorrectly classified as positive, *FN* (false negatives) presents the number of samples that are incorrectly classified as negative, *N* is the total number of samples, and *C* is the number of classes.

(3) Implementation Details

The proposed solution is evaluated through experiments on a workstation equipped with an Intel Xeon Gold 6226R CPU @ 2.90 GHz, and an NVIDIA RTX 3090 GPU (24 GB) running Ubuntu 22.04. The implementation is based on Python 3.9 and PyTorch 2.10 with CUDA 12.1. For the parameter tuning in our method and all baselines, we employ 4 layers for the encoder, set the dimension to 128, and the hidden dimension of feed-forward networks to 256. The model is trained using the Adam optimizer, with the learning rate initially set to 1 × 10^−4^ and a batch size of 32. Early stopping is employed during training, halting the process if the validation loss does not improve for 10 consecutive epochs to prevent overfitting. The training process runs for a maximum of 100 epochs, with model performance assessed based on classification accuracy, precision, and recall. Dropout regularization is applied at a rate of 0.1, while layer normalization is used throughout to maintain training stability and mitigate overfitting. To train and evaluate the model, the dataset is divided into training, validation, and test sets with a ratio of 7:2:1. To align with real-world data, measurement noise is introduced with a standard error of 0.1. For the efficiency evaluation, all models were measured on a single NVIDIA RTX 3090 GPU under an identical batch size and sequence length. Inference latency was recorded after GPU warm-up with device synchronization and averaged over 100 runs, and training time is reported per epoch. For a fair comparison, all baseline methods were re-implemented and evaluated on the same dataset as the proposed model, using identical training and test splits and the same evaluation protocol.

### 3.2. Quantitative Results Analysis

Accurate and real-time fault detection in photovoltaic systems is essential for early fault diagnosis and for ensuring reliable system operation. Table 3 presents the comparative performance of the proposed model and several mainstream time-series classification methods. All baseline models in this study are reproduced based on their official implementations. For clarity, the best results are highlighted in bold. As shown in Table 3, the proposed model consistently outperforms the compared baseline methods across all three evaluation metrics. Specifically, it achieves the highest classification accuracy of 88.4%, exceeding conventional sequence models such as recent transformer-based approaches, including Autoformer, Crossformer, Transformer, and iTransformer. This indicates that the proposed architecture is more effective in capturing discriminative patterns for photovoltaic fault classification. Among all methods, XGBoost, as a classical machine-learning baseline using hand-crafted statistical features, performs the worst (72.7% accuracy), indicating that such features cannot fully capture the temporal and inter-variable dependencies in PV fault signals. In terms of precision, the proposed model also attains the best performance (88.2%), demonstrating its superior capability in reducing false positives and providing more reliable fault identification results. Meanwhile, the F1 score reaches 87.3%, which is the highest among all compared methods, suggesting that the proposed model is more sensitive to fault occurrences and less likely to miss fault samples, which is an important property for early fault diagnosis in PV systems. Compared to S-Mamba, which already exhibits competitive performance among the baselines, the proposed model further enhances accuracy, precision, and recall. This performance gain highlights the effectiveness of the proposed dual-path modeling strategy in jointly capturing global temporal dependencies and variable-level dynamics. Overall, the results confirm that the proposed method provides a more balanced and robust classification performance for photovoltaic fault detection.

To evaluate computational efficiency, Table 4 compares the proposed model with the five baselines in terms of parameter count, training time, inference latency, and peak GPU memory. The three Transformer-based models incur substantial cost at the sequence length of 5000: the vanilla Transformer and Autoformer require 702.11 ms and 275.05 ms per inference and 171.25 GB and 10.52 GB of peak memory, respectively, reflecting the O(L^2^) complexity of self-attention. In contrast, the proposed model requires only 8.60 ms and 1.19 GB, reducing inference latency by one to two orders of magnitude. Compared with the lightweight single-channel models iTransformer and S-Mamba, the proposed model is slightly heavier (8.60 ms vs. 3.61 ms and 3.48 ms), as it employs two complementary encoding channels rather than one; this moderate overhead yields the consistent accuracy improvements reported above. As described in Section 2.1, fault diagnosis is performed on a 0.5 s sliding window, so the end-to-end detection delay is the window length plus the per-window inference time. Since the inference time is negligible relative to the window, the model meets the real-time requirement.

Table 5 compares the classification accuracy of different fault types across the evaluated methods, highlighting their performance variations under specific fault conditions. The proposed model achieves the highest accuracy in most fault categories, demonstrating its effectiveness across a wide range of operating scenarios. For fault types with relatively subtle or weak signatures, such as sensor faults and partial shading, the proposed model shows clear advantages over the baseline approaches. These fault types are typically characterized by low signal-to-noise ratios and complex temporal dependencies, making them more difficult to distinguish. The improved accuracy indicates that the proposed model is better at extracting discriminative temporal and cross-variable features from multivariate measurements. In the case of normal operation, the proposed model also attains the highest accuracy, suggesting improved stability in distinguishing healthy states from faulty conditions. For more severe faults, including open-circuit and short-circuit faults, all methods achieve relatively high accuracy due to the pronounced changes in system behavior. Even in these cases, the proposed model maintains a consistent performance edge. Furthermore, for grid-related disturbances and compound fault scenarios, such as grid anomaly combined with inverter fault, the proposed model remains competitive and delivers the best overall accuracy. This suggests that the model can effectively capture both local fault characteristics and broader system-level temporal patterns.

### 3.3. Visualization Analysis

**Figure 4:**
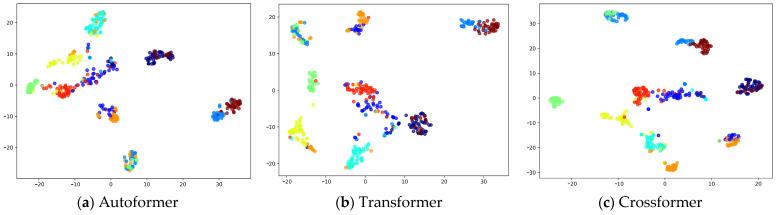

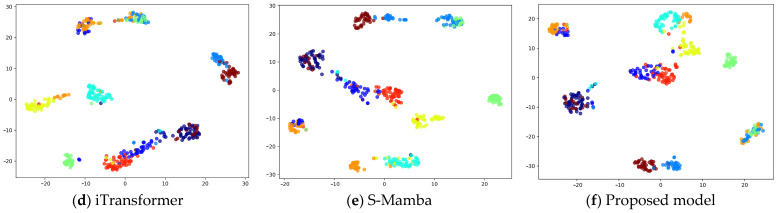
t-SNE visualization of feature representations learned by different models. Different colors denote different fault categories, and each point represents one sample in the t-SNE feature space.

To further investigate the discriminative capability of different models and gain insight into the feature representations learned for photovoltaic fault diagnosis, t-SNE is employed to visualize the extracted features in a low-dimensional space [46]. As observed in Figure 4, the learned feature distributions exhibit a clear progression in discriminative quality as the modeling capability of the compared methods improves.

As illustrated in Figure 4, the t-SNE visualizations reveal clear differences in the feature representations learned by different models. For Autoformer and the standard Transformer, samples from different fault categories exhibit noticeable overlap and scattered distributions, indicating limited class separability and insufficient discrimination between fault patterns. Crossformer and iTransformer show improved clustering behavior, with several fault categories forming more compact groups; however, partial overlaps and elongated clusters can still be observed, especially for faults with similar temporal characteristics. The S-Mamba model further enhances feature compactness, demonstrating clearer intra-class aggregation and reduced inter-class interference, which highlights the advantage of state-space modeling for long-sequence feature extraction. In contrast, the proposed model achieves the most distinct and well-separated clusters across all fault categories, with tight intra-class distributions and clear inter-class boundaries. This result indicates that the proposed dual-channel architecture effectively captures both global temporal dependencies and variable-wise dynamics, leading to more discriminative feature representations and improved fault separability. The visualization results are consistent with the quantitative performance gains reported in the classification experiments, further validating the effectiveness of the proposed method.

**Figure 5:**
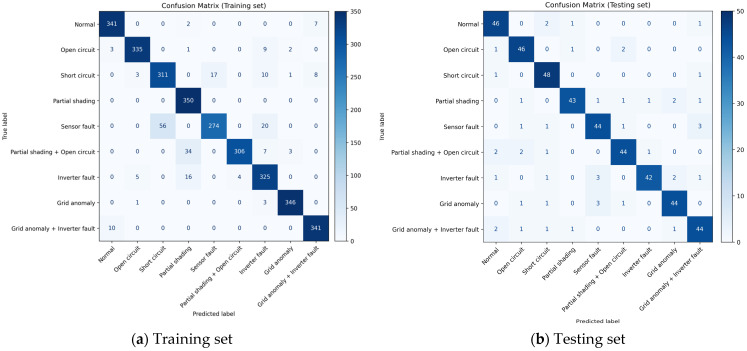
Confusion matrices of the proposed model on (**a**) the training set and (**b**) the test set.

To examine whether the model exhibits any bias across fault categories, Figure 5 presents the confusion matrices for the training and test sets. On the training set, almost all samples are correctly classified across the nine categories. On the test set, the predictions remain concentrated on the diagonal for every class, indicating balanced performance without systematic bias toward any particular category. The few misclassifications occur predominantly between physically related faults that share similar signatures, such as the inverter-fault, grid-anomaly, and sensor-fault classes. As the test set comprises only real-world measurements, these results demonstrate that the model generalizes in a balanced manner across all classes on real data.

## 4. Ablation Study

### 4.1. Ablation Study on the Dual-Channel Architecture

Table 6 reports the ablation results obtained by selectively enabling the time-embedding channel and the variable-embedding channel. When only the time-embedding channel is activated, the model achieves an accuracy of 78.1%, indicating that temporal modeling alone is insufficient to fully characterize the complex fault patterns in photovoltaic systems. Notably, although temporal modeling alone is insufficient as the dominant representation, an accuracy of 78.1% on a nine-class task is substantially above the chance level (≈11.1%), which confirms that the temporal token encodes genuinely discriminative fault information and functions as an active complementary feature rather than a regularization term. Enabling only the variable-embedding channel leads to a substantial performance improvement, with accuracy increasing to 87.1%, which highlights the importance of capturing variable-wise dependencies among multi-channel measurements. The best performance is achieved when both channels are jointly employed, yielding an accuracy of 88.4% and the highest F1-score of 87.3%. This result demonstrates that the two channels provide complementary information: temporal embedding enhances the modeling of long-term fault evolution, while variable embedding effectively captures inter-variable relationships. Their combination enables more discriminative feature representations and significantly improves fault diagnosis performance.

### 4.2. Impact of Embedding Methods on Model Performance

To investigate the effectiveness of the proposed dual-channel embedding strategy shown in Figure 2, an ablation study is conducted with different combinations of time embedding and variable embedding. As shown in Table 7, when the same embedding method is applied to both channels, i.e., using time embedding in both channels or variable embedding in both channels, the performance is noticeably degraded. Specifically, employing time embedding in both channels achieves an accuracy of 86.9%, while using variable embedding in both channels further reduces the accuracy to 84.7%. This indicates that a single embedding strategy is insufficient to simultaneously capture global temporal dependencies and variable-level dynamics.

When the embedding methods are mismatched, with variable embedding applied to the time channel and time embedding applied to the variable channel, the performance drops further, achieving an accuracy of 82.4%. This suggests that inappropriate alignment between embedding strategies and channel roles can significantly impair feature representation, as each channel is no longer optimized for the type of dependency it is intended to model.

In contrast, the proposed configuration, using time embedding in the temporal channel and variable embedding in the variable channel, achieves the best performance across all evaluation metrics. This result demonstrates that the proposed embedding design effectively complements the dual-path architecture by enabling each channel to focus on its intended modeling objective. From a structural perspective, these results confirm that time embedding is more suitable for capturing global temporal correlations across variables, while variable embedding is better aligned with modeling variable-specific temporal dynamics.

### 4.3. Impact of Different Encoders on Model Performance

To further clarify the role of encoder design in the dual-channel architecture shown in Figure 2, an ablation study is conducted by exchanging the encoders employed in the time-embedding and variable-embedding pathways. Specifically, the Frequency-Aware Temporal Module (FATM) encoder and the Bi-Mamba encoder are alternately assigned to the two channels, and the corresponding results are reported in Table 8.

When FATM is applied to both pathways, a noticeable performance degradation is observed. This indicates that the frequency-aware temporal modeling mechanism is effective for capturing global temporal characteristics but is less suitable for modeling variable-specific dynamics. Conversely, using Bi-Mamba in both channels improves performance but still fails to achieve optimal results, as the state-space-based modeling, while effective for long sequences, lacks explicit global token interaction along the temporal axis.

Further performance degradation is observed when the encoders are swapped, i.e., Bi-Mamba in the time channel and FATM in the variable channel, suggesting that misalignment between encoder inductive bias and channel functionality weakens feature representation. In contrast, the proposed configuration, FATM for the time channel and Bi-Mamba for the variable channel, achieves the best performance across all metrics. This confirms that assigning encoders according to their structural strengths enables complementary modeling of global temporal patterns and variable-level temporal dynamics, leading to more discriminative representations for fault classification.

## 5. Conclusions and Future Work

This work addresses the challenge of accurate and real-time fault detection in grid-connected photovoltaic systems by combining simulation-driven data generation with advanced time-series modeling. First, a grid-connected photovoltaic system simulation is established to generate a large amount of labeled fault data, in which diverse fault scenarios are systematically modeled under realistic operating conditions. Then, a Dual-Channel Enhanced Mamba Model is proposed for real-time fault detection in grid-connected photovoltaic systems, which decouples temporal modeling and variable-wise modeling into two dedicated channels, thereby effectively capturing global temporal dependencies and local intra-variable dynamics. Extensive experimental results demonstrate that the proposed approach consistently outperforms several mainstream time-series classification methods, while ablation and visualization analyses further validate the effectiveness and rationality of the dual-channel design. Overall, this work provides an effective and scalable solution for photovoltaic fault diagnosis under complex and dynamically changing conditions.

Future research will focus on extending the proposed framework to more complex operating scenarios, including a wider range of fault types and operating conditions, as well as enhancing the model’s adaptability to dynamically changing system behaviors. Additional sources of uncertainty, such as sensor drift, communication delays, and long-term system aging, will also be considered. Moreover, extending the proposed model toward fault localization and severity assessment, as well as exploring online or adaptive learning strategies, represents promising directions for enhancing its practical applicability in intelligent photovoltaic system monitoring.

## Figures and Tables

**Table 1:** Configuration parameters of the SW 325 XL module.

Module Parameters	Value
Number of cells	72
Open circuit voltage (*V*_OC_)	47.0 V
Short circuit current (*I*_SC_)	9.28 A
Maximum power point voltage	37.7 V
Maximum power point current	8.68 A
Series resistance	0.41 Ω
Shunt resistance	199.9 Ω
Temperature coefficient of *V*_OC_	−0.36%/°C
Temperature coefficient of *I*_SC_	0.102%/°C

**Table 2:** Description and characteristics of the different injected Faults.

Fault Side	Fault Type	Description
DC side	Open circuit	One or two parallel strings are disconnected by setting the open resistance (100–1000 Ω).
	Short circuit	Short-circuit faults are simulated by introducing a low-resistance path (0.01–0.1 Ω) between modules or between adjacent strings.
	Partial shading	Partial shading of the modules, ranging from 10% to 30%.
	Sensor fault	Current sensor faults due to poor contact or reading errors, with error magnitudes randomly set between 5% and 30%.
	Partial shading and Open circuit	Compound fault involving partial shading of modules and open-circuit disconnection in the PV array.
AC side	Inverter fault	Complete failure of one to three IGBTs in the three-phase inverter.
	Grid anomaly	Voltage sag or frequency anomalies caused by phase-to-phase or phase-to-ground faults in one or two phases of the system.
	Grid anomaly and Inverter fault	Concurrent grid anomalies combined with inverter faults lead to abnormal voltage or frequency behavior.

**Table 3:** Classification comparison with SOTA methods.

Methods	Accuracy (%)	Precision (%)	Recall (%)	F1 Score (%)
XGBoost [41]	72.7	73.5	72.8	73.1
Autoformer [42]	78.5	77.2	76.7	76.9
Transformer [43]	81.8	80.9	86.4	83.6
Crossformer [44]	83.0	83.8	84.6	84.2
iTransformer [45]	85.8	86.4	85.3	85.8
S-Mamba [40]	86.8	82.3	88.5	85.3
Proposed model	88.4	88.2	86.5	87.3

**Table 4:** Comparison of computational efficiency. All metrics are measured on a single NVIDIA RTX 3090 GPU with an identical batch.

Methods	Params (M)	FLOPs (G)	Training Time (s/epoch)	Latency (ms)	Memory (GB)
Autoformer	6.29	5.30	28.96	275.05	10.52
Transformer	6.30	5.34	122.43	702.11	171.25
Crossformer	4.72	4.07	20.67	49.01	7.75
iTransformer	1.18	0.02	3.39	3.61	0.06
S-Mamba	1.06	0.02	3.34	3.48	0.05
Proposed model	3.19	0.38	6.46	8.60	1.19

**Table 5:** Accuracy (%) comparison of different types across the methods.

Defect Types	Autoformer	Transformer	Crossformer	iTransformer	S-Mamba	Proposed Model
Normal	81.6	86.4	88.4	89.3	90.6	91.4
Open circuit	74.3	73.8	73.6	87.1	88.2	91.3
Short circuit	89.7	92.9	93.9	94.2	94.8	95.1
Partial shading	75.6	78.8	79.8	83.8	84.0	85.8
Partial shading and Open circuit	84.2	87.1	89.8	86.9	87.3	87.5
Sensor fault	69.9	78.8	79.2	82.6	84.4	87.2
Inverter fault	75.8	76.3	77.1	79.9	81.1	83.7
Grid anomaly	69.8	74.9	76.9	81.6	83.6	87.3
Grid anomaly and Inverter fault	84.9	87.4	88.2	87.3	87.3	87.6

**Table 6:** Ablation experiments of the dual-channel architecture.

Time Embedding Channel	Variable Embedding Channel	Accuracy (%)	Precision (%)	Recall (%)	F1 Score (%)
√		78.1	81.8	78.7	80.2
	√	87.1	86.6	87.3	86.9
√	√	88.4	88.2	86.5	87.3

**Table 7:** Ablation study of the embedding methods.

Channel	Time Embedding Channel	Variable Embedding Channel	Accuracy (%)	Precision (%)	Recall (%)	F1 Score (%)
Embedding methods	Time embedding	Time embedding	86.9	85.8	88.7	87.2
	Variable embedding	Variable embedding	84.7	84.6	82.3	83.4
	Variable embedding	Time embedding	82.4	81.8	86.2	83.9
	Time embedding	Variable embedding	88.4	88.2	86.5	87.3

**Table 8:** Ablation study of different encoders.

Channel	Time Embedding Channel	Variable Embedding Channel	Accuracy (%)	Precision (%)	Recall (%)	F1 Score (%)
Encoder	Bi-Mamba	FATM	87.9	86.2	87.9	87.0
	FATM	FATM	85.6	83.2	85.2	84.2
	Bi-Mamba	Bi-Mamba	88.1	82.7	87.8	85.2
	FATM	Bi-Mamba	88.4	88.2	86.5	87.3

## Data Availability

The data presented in this study are available on request from the corresponding author.
